# Coupling of droplet-on-demand microfluidcs with ESI/MS to study single-cell catalysis†

**DOI:** 10.1039/d4ra04835k

**Published:** 2024-08-13

**Authors:** Marie van der Loh, Marie Schiffmann, Matthias Polack, Konstantin Wink, Detlev Belder

**Affiliations:** a Institute of Analytical Chemistry, Leipzig University Linnéstraße 3 04103 Leipzig Germany belder@uni-leipzig.de

## Abstract

Droplet microfluidics provides an efficient method for analysing reactions within the range of nanoliters to picoliters. However, the sensitive, label-free and versatile detection with ESI/MS poses some difficulties. One challenge is the difficult association of droplets with the MS signal in high-throughput droplet analysis. Hence, a droplet-on-demand system for the generation of a few droplets can address this and other problems such as surfactant concentration or cross-contamination. Accordingly, the system has been further developed for online coupling with ESI/MS. To achieve this, we developed a setup enabling on-demand droplet generation by hydrodynamic gating, with downstream microscopic droplet detection and MS analysis. This facilitated the incorporation of 1–9 yeast cells into individual 1–5 nL droplets and the monitoring of yeast-catalysed transformation from ketoester to ethyl-3-hydroxybutyrate by MS. With our method a mean production rate of 0.035 ± 0.017 fmol per cell per h was observed with a detection limit of 0.30 μM. In conclusion, our droplet-on-demand method is a versatile and advantageous tool for cell encapsulation in droplets, droplet imaging and reaction detection using ESI/MS.

## Abstract 2

Glass chips were implemented to facilitate the coupling of a droplet-on-demand system with ESI/MS, thereby enabling the detection of reactions catalysed by a single yeast cell.

## Graphical abstract

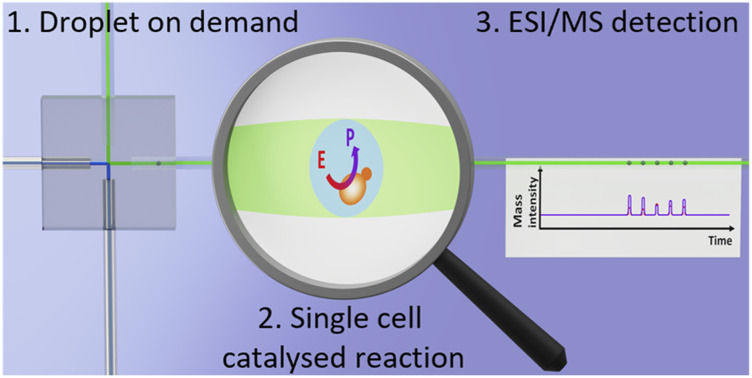

## Introduction

Droplet microfluidics is one of the most successful microfluidic technologies, particularly for high throughput screening (HTS). The technology has matured over years and gone beyond academia with applications in biomedicine, biotechnology, and material science, cosmetics, food, and pharmaceuticals.^1–8^

An active area of research continues to be the coupling of droplet microfluidics with mass spectrometry (MS) in order to detect the chemical content of μl- to nl-sized droplets with unrivalled chemical information value. Droplet microfluidics has been coupled to MS mainly by the following methods: ESI/MS^6,9–34^ or by MALDI/MS.^35–40^

In almost all of these areas of droplet-MS coupling, the generation of aqueous droplets in a continuous oil phase was achieved through passive generation.^41^ Thereby, the aqueous and oil phases are continuously pumped together, resulting in the formation of a segmented phase in a junction region.^3^ In the oil phase, the aqueous droplets are usually arranged like pearls on a string. The highest frequency measured for droplet introduction in the MS is above 30 Hz.^30^ This approach is therefore robust and has been described as an efficient method for coupling to mass spectrometry using an online Electrospray ionisation interface.

An alternative to passive droplet generation is active generation, (also known as droplet-on-demand, DoD) focusing on the controlled generation of a few selected droplets. In contrast to the aforementioned approach, the entire discontinuous phase is not continuously converted into droplets, but rather a specific portion at a specific time. This attractive feature allows the selection and tracking of individual species, such as cells, within the phase to be dispersed. The optical triggering of droplet production based on the presence of cells is particularly advantageous.^42–44^

Active droplet generators can be designed through various means.^45^ These include active generators manipulating additional forces, such as electrical or magnetic, or inherent forces influenced by material properties or fluid viscosities.^3,46–48^

Compared to passive droplet generation, the coupling of droplet-on-demand systems with online ESI-MS is more challenging for several reasons. For example, the continuous phase predominates in the spraying process or the outlet of the segmented flow is occupied by the MS interface functionality. Therefore, the outlet cannot be used to control active droplet dosing, *e.g.* by conventional vacuum pulses.^42^ Furthermore, additional forces are not always compatible with cells.^49^

Accordingly, the coupling has only been described in a few publications, including that of Teixidor *et al.*, in which 30–60 nL droplets were generated after prolonged contact with cerebrospinal fluid and subsequently stored in PFA capillaries.^50^ The work by Sun *et al.* involved active droplet generation using valves and subsequent ESI/MS analysis.^51^ In this approach, the droplets were extracted in ESI-compatible liquid and sprayed separately from the oil.

In a previous work, we developed an approach to monitor single cell catalysis by droplet microfluidics-MS coupling using standard passive droplet generation.^34,52^ In the current study, we are now exploring a compatible droplet-on-demand technology for this application scenario. By avoiding close spacing between droplets, the previous problem of droplet coalescence at low surfactant concentrations to achieve high MS sensitivity will be circumvented. In addition, storage of the droplets in the channels or capillaries is much easier than with passive droplet generation, which should allow more precise microscopy of the droplets for counting and inspection of the entrapped cells.

## Experimental

The chemicals employed in this study were procured from Sigma-Aldrich unless otherwise stated. The oils Novec 7500 and Fluorinert™ FC-40 were obtained from IOLITEC Ionic Liquids Technologies GmbH, and caffeine was sourced from Honeywell Fluka. For the yeast-catalysed reaction, commercially available yeast (Dr August Oetker KG) was acquired and prepared following the procedure outlined in Wink *et al.*'s publication with 0.6 mM glucose in 10 mM ammoniumacetate.^34^ A target cell count of 0.1 optical density at 600 nm (OD_600_) was sought and adjusted through appropriate dilution.

A variety of materials were considered for hydrodynamic gating, as shown in Fig. S1† in-house fabricated polydimethylsiloxane (PDMS), and fused-silica glass chips manufactured *via* selective laser-induced etching (SLE) and subsequent wet-chemical etching in KOH as described in Heiligenthal *et al.*'s work,^33^ with channel diameters ranging from approximately 50 to 100 ± 15 μm. PDMS chip fabrication was carried out using standard soft lithography techniques.^31^ While PDMS chips allowed for direct droplet storage, glass chips required post-processing. In this case, HPFA capillaries (OD 360 μm, ID 100 μm, Postnova) were affixed to the glass chip using epoxy resin (Epoxy technology) to create hydrophobic storage channels (shown in Fig. S1†). Before usage, the glass chips were subjected to a hydrophobisation process. The glass chip was initially rinsed with dry isooctane (Merck KGaA, dried over a molecular sieve with a pore-size of 3 Å), followed by a slow rinsing with trichloro-(1*H*,1*H*,2*H*,2*H*-perfluorooctyl)silane dissolved in dry isooctane. Subsequently, it was once again rinsed with dry isooctane and purged with nitrogen. It is essential to prevent moisture from entering the channels during the silanisation process. To enhance the hydrophobicity of the surfaces, both the silanised glass and PDMS chips were treated with Rain Repellent (Rain-X) for 10 minutes and subsequently rinsed with isopropanol.

The liquid flows were generated using Fluigent's Line-Up pressure-driven pumps. Fluigent's All in One and Microfluidic Automation Tool (MAT) scripting programs were used for the precise control of the pumps. The resulting droplet dimensions were determined using ImageJ software (see Fig. S3†).

For the yeast-catalysed reaction, the setup has been modified to ensure that the reactants only come into contact after a PEEK T-cross (VICI, bore: 100 μm), as shown in Fig. S10.† The droplets were generated in glass chips immediately after the reactants 40 μM ethyl-acetoacetate (EAA, 20 μM in the droplet) with the 10 μM internal standard ethyl-4-chloro-3-hydroxybutyrate (EClHB) and the yeast solution were mixed. The resulting droplets were collected in HPFA capillaries (refer to Fig. S12†) and held at room temperature for approx. 20 hours. The droplets were visualised using microscopic methods with a microscope (Olympus, IX70) and a portable microscope as used in prior publications.^53,54^

After storage, the droplets were subjected to ESI/MS for analyte detection, employing an Agilent CE coaxial sprayer (trible tube sprayer). The MS parameters operated on the Agilent TQ 6495B mass spectrometer are dwell time 2 ms, nebuliser 5 psi and sheath liquid consisting of MeOH/H_2_O (1 : 1) and 0.1% formic acid at a flow rate of 5 μL min^−1^ and are listed in more detail in Table S2.† The droplets were brought into contact and ionised at the emitter end within the sheath liquid environment. Analytes, including caffeine (Percursor-ion *m*/*z* 195.0 → Product-ion *m*/*z* 138.0, collision energy (CE) 25 V), ethyl-3-hydroxybutyrate (EHB, *m*/*z* 133.1 → *m*/*z* 87.1, CE 7 V), ethyl-acetoacetate (EAA, *m*/*z* 131.1 → *m*/*z* 85.1, CE 7 V), and the internal standard ethyl-4-chloro-3-hydroxybutyrate (EClHB, *m*/*z* 167.1 → *m*/*z* 121.1, CE 7 V), were detected in positive mode using the multiple reaction monitoring (MRM) mode. Syringe pump-driven transport (neMESYS, Cetoni) at a flow rate of 0.2 μL min^−1^ facilitated the delivery of droplets containing yeast cells to the emitter.

## Results and discussion

We developed an active droplet generation method employing a hydrodynamic gating mechanism using pressure alterations and flow modulation at the cross-junction channels to avoid vacuum pulses at the outlet.

The hydrodynamic gating approach has been successfully applied in pre-tests to various discont. phases such as water and acetonitrile (ACN), as well as cont. phases such as Novec 7500 and FC-40. The compatibility of the DoD method with chip materials, namely PDMS and glass, is discussed in the following. PDMS was chosen to optimise hydrodynamic gating due to its rapid fabrication capabilities, allowing efficient exploration of different modifications. In addition, in previous studies, PDMS has been the primary material for DoD fabrication,^3,45,55^ offering advantages such as hydrophobicity,^56^ seamless coupling with ESI/MS,^11,31^ and oxygen permeability.^57^ However, it has been shown to be unsuitable for prolonged droplet storage in the context of cellular reactions.

In contrast, fused-silica glass chips produced by SLE offer flexibility for rapid prototyping and are more suitable for a semi-automatic mass production than PDMS. These glass chips have improved rigidity and chemical resistance.^56^ To make the inherently hydrophilic glass surface hydrophobic, a surface modification with silane and RainX was used. This treatment, combined with hydrophobic HPFA capillaries, ensures reliable droplet formation and transfer to storage. Furthermore, HPFA capillaries offer several advantages, including extended droplet storage within sealed capillaries and a seamless connection to the MS without the need for surfactants.

**Fig. 1:**
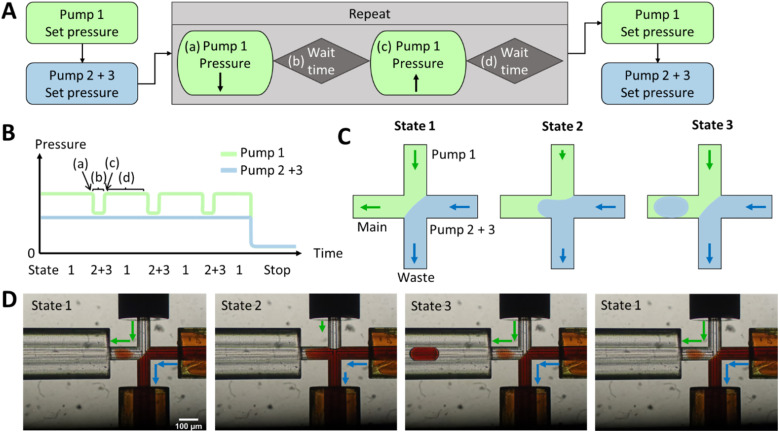
(A–C) A schematic illustration of the script programmed into the MAT software. (D) The microscopic images illustrate the various states through the use of a coloured water phase containing Fe(SCN)_3_. Initially, state 1 is established, followed by the configuration of automated pressure pulsing. During this process, the pressure of pump 1 (cont. phase) is decreased (a). Subsequently, after a defined waiting period (b), the pressure of pump 1 is returned to its initial value (c). Then, after another waiting period (d), multiple cycles can be executed to generate additional droplets, as seen in states 2 and 3. Finally, the pump pressures can be adjusted differently, such as setting them to ambient pressure to cease droplet generation.

The hydrodynamic gating automated script for droplet generation with the Fluigent LineUp series is described in Fig. 1 and S2,† where in Fig. 1 three pumps are used for reaction mixing and in Fig. S2† only two pumps are used for pre-testing and seamless connection to ESI/MS. Initially, pressure was applied to the reservoirs (as shown in Fig. S9†), forcing the liquid into the chip. A lower pressure for the discont. phase (in this case, dionised water) was required to prevent occasional droplet formation and to maintain a stationary condition. The discont. phase was directed to the waste, while the cont. phase mainly flowed into the main channel but could also enter the waste in minor amounts. However, for online MS detection, a constant flow towards the MS was critical for a stable Electrospray and determining the noise levels.

Once the interface between the discont. and cont. phases was established, the script for the pressure pulses was initiated. If only one pump is required for the discont. phase and no prior pumping and mixing of two different liquids is necessary, then the pressure pulses can be applied to the cont. phase, as it can be seen in Fig. S2.†

The droplet size variation and a parameter screening were performed in PDMS chips and placed in the ESI.† Parameters such as pressure intensity, waiting time *b* and pressure pulse intensity were tested in PDMS chips with 10 to 20 pulse repetitions (see Fig. S1A†). It was found that a pressure of 300 mbar was sufficient to obtain a stable interphase between the discont. and cont. phases (see Fig. S1B and S6†). Fewer droplets were produced at lower pressures, and the droplet size increased. Over 400 mbar, even smaller droplets were produced, and a droplet formed with almost every pulse. However, at higher pressures, the flow is faster and unsuitable for direct coupling.

The pressure increases of 5–20 mbar were analysed for the pulse intensity parameter (see Fig. S4†). The lower the intensity, the smaller the droplets. It was observed that droplets with a diameter smaller than the inner diameter of the channel adhered to the PDMS wall. At higher pulse intensities, 2–3 droplets were produced in most cases, with some droplets often merging. These two effects resulted in droplets of different volumes, which in turn increased the standard deviation. The pulse intensity should, therefore, be set in the range of 5–10 mbar for PDMS chips so that only one droplet is produced per pulse. The most successful setting was found to be 8 mbar.

The waiting time did not affect the size of the droplets (shown in Fig. S5†). Nevertheless, no droplets were produced per pulse if the waiting time was too short, *e.g.*, 100 ms. At 1 s, several droplets could form in that time, resulting in an increased standard deviation due to droplet coalescence. Therefore, a 200–500 ms waiting time is sufficient to generate droplets and reduce multiple droplet formation.

New pressure ratios had to be applied to the glass chips as the back pressure had changed. Therefore, a parameter screening of intensity and waiting time was repeated in glass chips (as shown in Fig. S7†). Moreover, a chip-to-chip analysis was conducted on two glass chips under the same conditions with different waste capillaries as illustrated in Fig. S8.† The mean droplet size values exhibited no significant difference between the two chips, however the standard deviation was found to be higher for one chip than the other. Additionally, the droplet size demonstrated greater variation for the glass chips than for the PDMS chips. It was determined that the capillary length and potential imperfections in the bonding of the capillary, respectively, resulted in increased pressure fluctuations. Volume variation due to changes in hydrophilicity may also be contributed by an attached droplet. A more stable flow could be achieved at a higher back pressure, *e.g.* by using capillaries with a smaller inner diameter. An alternative approach was explored through the utilisation of flow sensors. However, the introduction of this additional functionality hampered the ability to fine-tune the droplet generation.

For the yeast-catalysed reaction in the droplet, however, the cont. phase was reduced by pressure pulses in glass chips. Two pressure-based pumps were used for the discont. phase of two reactants, as shown in Fig. 1. These were adjusted as before to create an equilibrium between the discont. and cont. phases. The pressure of the cont. phase was reduced (shown in Fig. 1A), maintained for the waiting time *b*, and then increased by the same amount. Waiting time *d* was adjusted to allow the system to regain stability (shown in Table S1†). The previously set equilibrium was typically restored in less than 1 s. A cycle was incorporated to create a new droplet. Therefore, 1 Hz has been used here to achieve good droplet spacing and to ensure that each droplet can be detected in the MS. However, it is possible to set a higher droplet generation frequency through the waiting time *d* and the flow rate, but care must be taken to adjust the stability of the interface (in a few 100 ms as shown in the Video 1†). After droplet formation, the programme could either stop droplet production or set a different pressure to reduce the flow rate. The yeast is encapsulated in droplets according to Poisson's distribution.^58^ In the future, this pre-imaging should facilitate the directed triggering of droplet generation and encapsulation of single cells.^42^

Different starting pressures had to be set depending on the chip dimensions (shown in Fig. S7B†). Thereby, pulse durations ranged from 20 ms to 1 s, with intervals between pulses of 1–5 s (shown in Fig. S7A†). Table S1 in the ESI† provides an example script from the Fluigent MAT program.

**Fig. 2:**
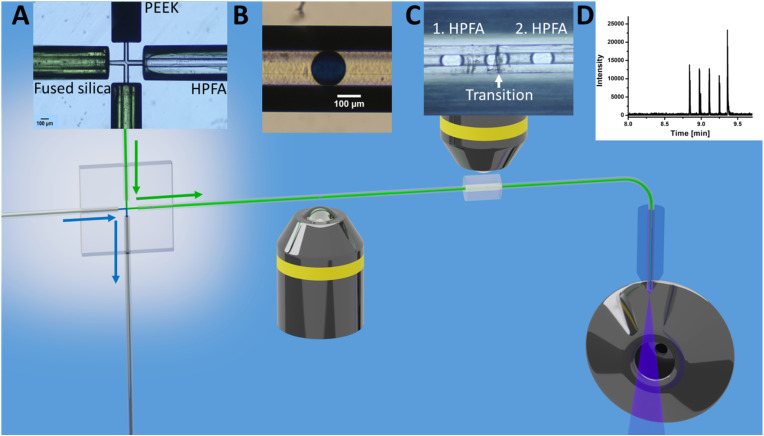
Schematic of the steps for direct coupling of active droplet generation to ESI/MS of caffeine droplets. (A) droplet generation; (B) droplet imaging and storage; (C) transition of droplets and (D) mass spectrometric detection. Each step was observed under the microscope and reflected the respective stage's image. The channels at the cross in A had a diameter of 60 μm and the introduced capillaries was fused silica, PEEK and HPFA.

As shown in Fig. 1D, a pressure pulse aimed to produce one droplet per pulse. Therefore, the pressure was carefully adjusted to maintain a balance that ensured the production of uniform droplets in the 1–5 nL size range per pulse without excessive droplet formation. For the subsequent experiments, the glass chip was seamlessly connected to the ESI/MS. For purposes of clarity, Fig. 2 illustrates the setup for direct coupling of DoD to ESI/MS, with the actual setup shown in Fig. S11.† The discont. phase (shown in blue) flows alongside the cont. phase (shown in green) at the cross-junction in Fig. 2A. In Fig. 2B, the actively formed droplets are subsequently identified using a portable microscope. After transfer to the emitter capillary *via* a transition capillary (shown in Fig. 2C), the droplet is sprayed with the trible-tube sprayer and analysed in the MS (shown in Fig. 2D).

**Fig. 3:**
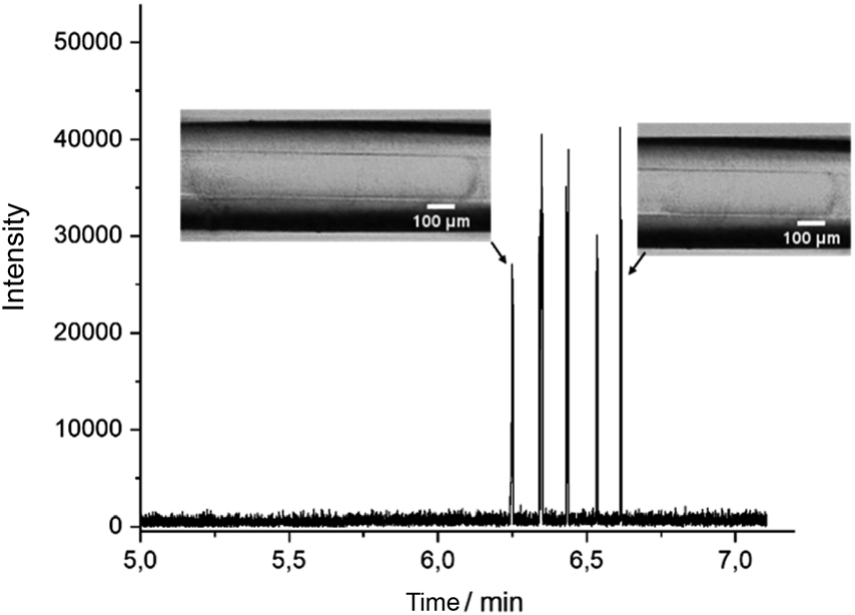
Mass trace of DoD generated water droplets with 10 μM caffeine with direct mass spectrometric coupling through the connector in MRM mode. Cont. phase: FC-40; sheath liquid: MeOH/H_2_O + 0.1% FA. Droplet size from first MS signal 7.2 nL, 10.1 nL; 5.2 nL 3.0 nL to last signal 4.3 nL.

DoD was performed for five pressure pulses using the MAT system from Fluigent pressured driven pumps. A 10 μM caffeine solution in water was used as the test substance in the discont. phase. The droplet was then stopped in the HPFA capillaries. After a period of time, the liquid flow was restarted with a reduction to improve the peak shape, where the pressure of the discontinuous phase is lower. Consequently, five signals were expected to be visible in the mass trace in Fig. 3. The width of the MS signal is comparable to the corresponding droplet size (shown in the appendix in Fig. S13†). This seamless connection between the droplet generator and the ESI/MS is particularly advantageous for enabling and detecting a rapid reaction. It provides an online ESI/MS setup for active droplet generation. Notably, the droplets were not stabilised with a surfactant, as would be required with stainless steel capillaries or without oil spacing. The use of a triple tube sprayer eliminates the need for prior extraction.

A small section of HPFA capillary, as depicted in Fig. S12,† was employed to accommodate the incubation of multiple droplets side by side. This setup provides the advantage of observing and assessing droplets before deciding whether to retain or produce new ones. Longer reactions can be monitored and evaluated.

In the context of a longer cell-catalysed reaction within the droplets, yeast cells were transferred into a 10 mM ammonium acetate solution containing 0.6 mM glucose. The buffer has been chosen to facilitate the measurement for ESI/MS. To ensure that each droplet contained at least one cell as described by the Poisson distribution,^58^ the optical density (OD600) was adjusted to 0.14. The precise enumeration of cells within each droplet occurred during storage inside the HPFA capillary. An optical examination of the standing droplet was carried out, enabling accurate microscopic observation involving both horizontal observations using a microscope (shown in Fig. 5B) and lateral observations using a portable microscope. The findings revealed that some yeast cells settled at the bottom of the droplet after a few seconds, which could lead to their oversight, particularly when focusing on the middle part of the channel. This DoD approach, followed by storing the resting droplets, allows close examination of the cells (shown in Video S2†), ensuring that no cell is missed. In addition, smaller microorganisms such as *E. coli* can be observed at the single cell level in the droplet using high magnification objectives. The DoD is therefore more suited to one of the imaging challenges of visualising cells in droplets in a flow.

**Fig. 4:**
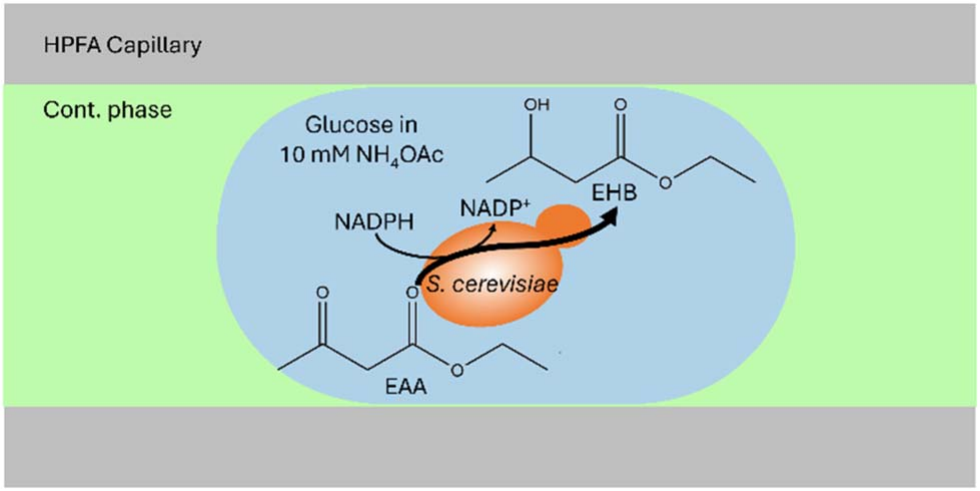
Schematic illustration of the yeast-catalysed reaction in an aqueous droplet (in blue) surrounded by the cont. phase (in green) in a HPFA capillary. Within the droplet, EAA is converted to EHB, for which at least one baker's yeast must be present in the droplet.

**Fig. 5:**
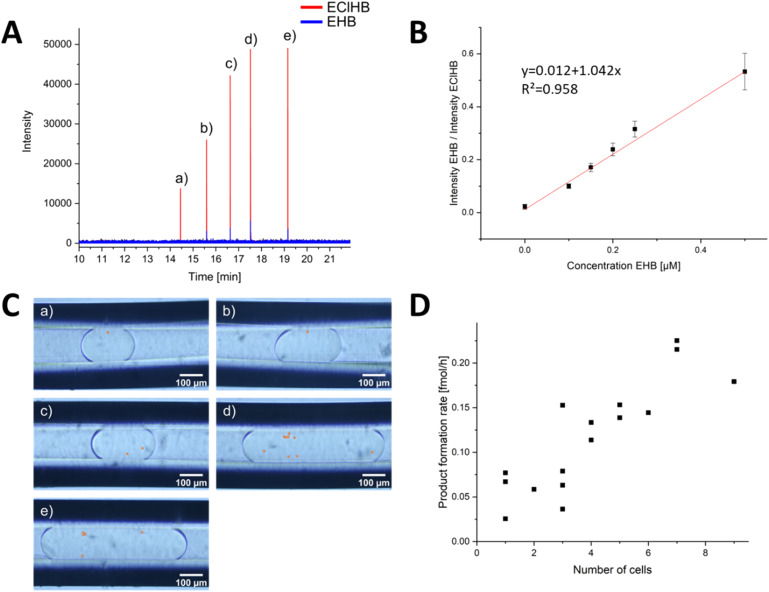
Evaluation of the DoD-generated droplets by ESI/MS. (A) mass trace of 5 droplets in MRM mode, each generated by one pressure pulse. The distribution of cells in the droplets is statistically distributed. The droplets of the corresponding signals are shown in (C). The number of cells (highlighted in orange) in the droplets can be counted. The calibration series in (B) was used for the evaluation. Each point in the calibration was obtained from a minimum of 31 droplets in MRM mode, and a Grubbs outlier test was performed to detect outliers. In (D), the product formation rate for each cell number is shown. In addition, the droplets in Fig. S15 and S16† from other storage capillaries were included.

After the successful development of a DoD system coupled with ESI/MS, we extended the methodology to the study of whole-cell catalysis using the model system exemplified by the reduction of ethyl acetoacetate (EAA) to ethyl-(R)-3-hydroxybutyrate (EHB), shown in Fig. 4. This reaction, which has been extensively documented in previous publications,^59–62^ serves as a model for the production of chiral alcohol precursors that are important in the pharmaceutical, flavour and fragrance industries.^59,62,63^ The process involves the use of a carbonyl reductase enzyme within yeast, with NADPH acting as a cofactor for the reduction.^61,63^ The use of yeast as a whole-cell catalyst is advantageous because of its inherent regeneration of the cofactor. This regeneration occurs by converting the reduced form to the oxidised form within the pathway.^61,63^ According to Houng, glucose is the most suitable substrate for this purpose.^62^ An additional advantage is using readily available baker's yeast from supermarkets.^62^ In the following the successful encapsulation of yeast in DoD droplets the reaction was carried out in the aqueous phase. Microscopic observations were then made to determine the amount of yeast within the droplets, as shown in the microscopic images in Fig. 5B

To mix the reagent solutions, a PEEK T-cross (depicted in Fig. S10†) was incorporated ahead of the chip. The reduction of a ketoester to EHB was traced *via* mass spectrometric detection. The product EHB was quantified using the internal standard ethyl-4-chloro-3-hydroxybutyrate (EClHB). Each calibration data point in Fig. 5B corresponds to MS signals from at least 31 droplets (evaluation shown in SI). These droplets of different sizes were then transferred sequentially for MS detection. The limit of detection (LOD), calculated as recommended by the International Committee on Harmonization (see ESI† for detailed information), was determined to be 0.30 μM, a value comparable to the LOD of 0.25 μM reported in the publication by Wink *et al.*^34^ The fluctuations in the Electrospray were corrected using the internal standard.

In order to identify each droplet, the DoD system was modified to generate only 5 droplets, which were then transferred to a separate capillary using a controlled flow. The number of yeast cells within these droplets was promptly determined, as shown in Fig. 5C. After a 20 hours reaction time, the storage capillary was connected to the MS, and the flow was initiated slowly. Bulk experiments by Wink *et al.* have shown that the reaction is complete after 22 hours.^34^ The droplets were again observed under a portable microscope (shown in Fig. S11†). No excessive increase in the number of yeast cells was observed, confirming that no yeast cultivation occurred. This is primarily because the growth medium lacks the necessary nutrients for yeast proliferation and is designed solely to sustain yeast metabolic reactions.^34,64^ This aspect is particularly crucial for meaningful single-cell analyses, ensuring that no additional cells are measured.

The droplets were subsequently directed into a triple-tube sprayer at a flow rate of 0.2 μL min^−1^ and sprayed with a sheath liquid. First, droplet splitting occurred due to electrowetting without grounding the PFA emitter capillary, a phenomenon previously described in Peretzki's publication for glass chips.^65^ After grounding, no splitting was observed, and as depicted in Fig. 5A, each droplet was individually resolved in the mass trace. By employing the internal standard EClHB, the variations in spray characteristics and the influence by the glucose and other accompanying substances causing differential signal intensities were compensated, enabling quantification using the calibration curve in Fig. 5B.

The obtained results demonstrated variations linked to cell activity and the number of cells encapsulated within individual droplets. Fig. 5D highlights that a higher cell count resulted in increased product formation. Notably, the product concentration consistently surpassed the LOD for all yeast-cell-containing droplets, affirming the occurrence of cell-catalysed reactions within the DoD-generated droplets. As depicted in Fig. S17,† there was no correlation between droplet volume and EHB concentration. While the LOQ of 0.91 μM remained unattained in certain droplets and approached closely in others, the lowest concentration recorded for a droplet with one cell was 0.31 μM at 1.6 nL droplet volume.

The mean product formation rate observed herein, amounting to 0.035 ± 0.017 fmol per cell per h, represents a lower rate compared to that reported by Wink *et al.*^34,66^ The observed variability in the standard deviation can be attributed to the inherent heterogeneity among the cells. Consequently, the high-producing cells were not included in the analysis due to the limited number of droplets. However, a variation in the number of cells in the droplets was detected, with a clear correlation between the number of cells and the MS signal.

## Conclusions

As demonstrated in this study, the use of active droplet generation by hydrodynamic gating is proving to be a versatile and advantageous method compared to passive generation techniques. The main advantages of the droplet-on-demand method are that it is easy to implement, compatible with different materials and solvents and has a high biocompatibility. Additionally, no surfactant is needed, and the MS signal can be assigned to each droplet. In this approach, pressures were strategically applied at the inlets to induce droplet formation *via* pressure pulses. The number of droplets and their size can be influenced by a number of factors, including pressure fluctuations, an increase in the intensity of the pressure pulse and the hydrophobicity of the channel surface. Detection was seamlessly integrated from a glass chip coupled to ESI/MS *via* a HPFA capillary. As an example, the analysis of 1–5 nL droplets containing 1–9 yeast cells each was performed. The ability to monitor the reaction products of cell-catalysed reactions by ESI/MS was successfully demonstrated.

This hydrodynamic gating approach, coupled with ESI/MS, is auspicious in scenarios involving a limited number of cells or when the focus is on single droplet analysis. It greatly facilitates the establishment of correlations between droplets and MS signals, thus providing a valuable tool for validating reactions within droplets.

A future application is the implementation of triggered DoD by cells for single-cell encapsulation, as proposed by Chen *et al.*^42,43^ In addition, manipulation strategies such as splitting and sorting, as in mass-activated droplet sorting (MADS),^32,67^ can be integrated with the DoD system. This integration aims to improve the assignment of smaller volumes, opening up avenues for refined experimentation and analysis in microfluidic environments.

## Data availability

Data for this article, including MS raw data, MATLAB code and additional pictures and videos are available at RADAR4Chem at https://doi.org/10.22000/VibgsJCOWdWhxqgl.

## Author contributions

M. v. d. L. visualisation, writing – original draft, M. v. d. L. and M.S. experimental, formal analysis, validation, writing – review & editing M. P. conceptualisation and creating of the glass chips. Writing – review & editing; K. W. conceptualisation of the reaction, writing – review & editing; D. B. conceptualisation, supervision, project administration, writing – review & editing.

## Conflicts of interest

There are no conflicts to declare.

## Supplementary Material

RA-014-D4RA04835K-s001

RA-014-D4RA04835K-s002

RA-014-D4RA04835K-s003

RA-014-D4RA04835K-s004
