# Supplementary material for: Coupling of droplet-on-demand microfluidcs with ESI/MS to study single-cell catalysis
Source: RSC Adv. 2024 Aug 13;14(35):25337–46. doi: 10.1039/d4ra04835k (PMC11320962; doi:10.1039/d4ra04835k)
Supplement: RA-014-D4RA04835K-s001 [file RA-014-D4RA04835K-s001.pdf]

Supplementary Information (SI) for RSC Advances.
This journal is © The Royal Society of Chemistry 2024

Supplementary Information

Coupling of Droplet-on-Demand Microfluidcs with ESI/MS to Study Single-Cell Catalysis

Marie van der Loh, [#a] Marie Schiffmann,[#a] Matthias Polack,[a] Konstantin Wink [a] and Detlev Belder *[a]

Table of contents

Comparison of PDMS and glass chips 1
Method for Droplet on Demand generation with pressure-driven pumps 2
Droplet volume calculation 3
Droplet on demand parameter screening 4
Setup for the generation of droplets 6
Setup of a glass chip or HPFA capillary for MS measurements 7
Storage capillary 8
Mass trace evaluation 9
Calculation of the LOD and LOQ 10
Mass traces of additional capillaries with droplets 10
Droplet Generation Videos 13
Droplet Examination Video 13
Capillary Connection Video 13
References 13

Comparison of PDMS and glass chips

We have investigated different materials for droplet on demand (DoD) generation as shown in Figure S 1. The dimensions of the PDMS chips are approx. 100 µm in height and width in rectangular channels. At the junction, the width is reduced to about 50 µm. On the other hand, the SLE-manufactured fused-silica glass chip has cylindrical channels with a diameter of approx. 100 µm. The capillaries used also have an internal diameter of 100 µm. The capillaries were glued into the chips with epoxy glue so that the ends were directly connected to the channels, as shown in Figure S 1.

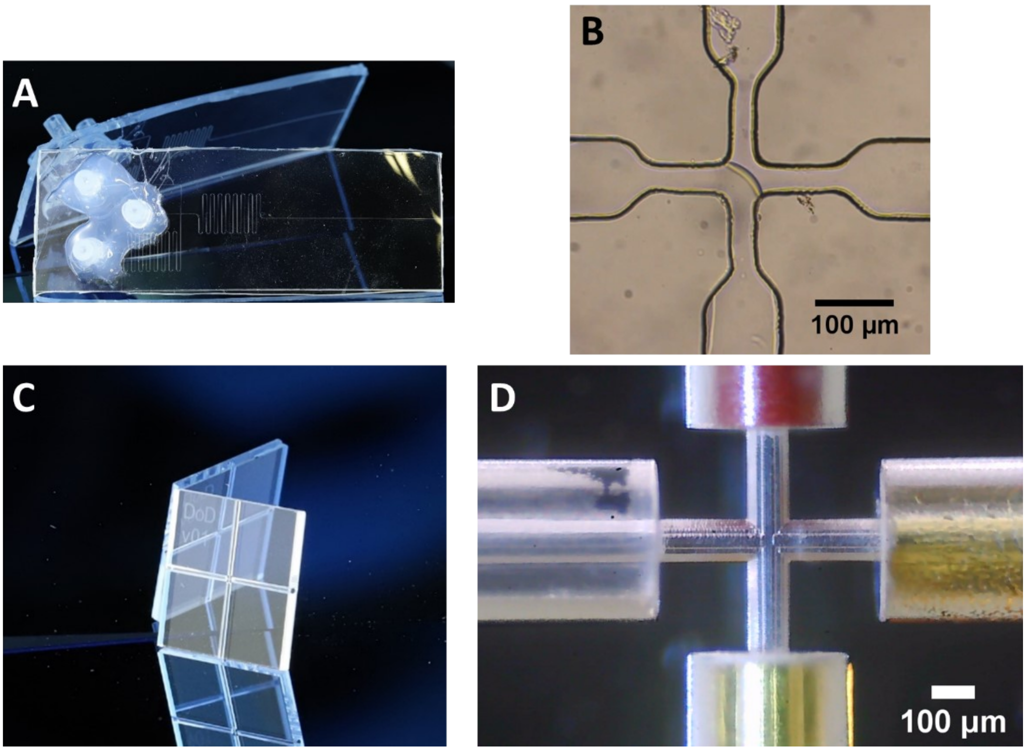

Figure S 1: Photographic images of A: PDMS chip and C: glass chip and a microscopic image of the junction in B: PDMS chip with water and FC-40 in a stable interphase and D: glass chip with capillaries glued inside the chip: PEEK capillary on top, fused silica glass capillaries on the right and bottom and HPFA capillary on the left.

Method for Droplet on Demand generation with pressure-driven pumps

To initiate droplet formation, an interface between the discontinuous and continuous phases was meticulously created at the cross junction. To achieve this, precise pressure settings were configured using Fluigent's A-i-O 2019 software. Appropriate pressure pulse parameters were then carefully selected and fine-tuned to produce single droplets, as shown in Figure S2.

During the preliminary experiments, the pulse pressure was increased to the aqueous phase (pump 2). As shown in Figure 2 and Figure S4, two different aqueous phases were used in the reaction. It was observed that increasing the pressure in both phases could potentially have a delayed effect on droplet formation. Consequently, a strategic decision was made to reduce the pulse pressure in the continuous phase. The control program used for this purpose is the Microfluidics Automation Tool 2019, with the script provided in Table S1. Notably, this pressure reduction had previously been successfully tested on polydimethylsiloxane (PDMS), although the results are not presented here and have now been seamlessly adapted to glass chips.

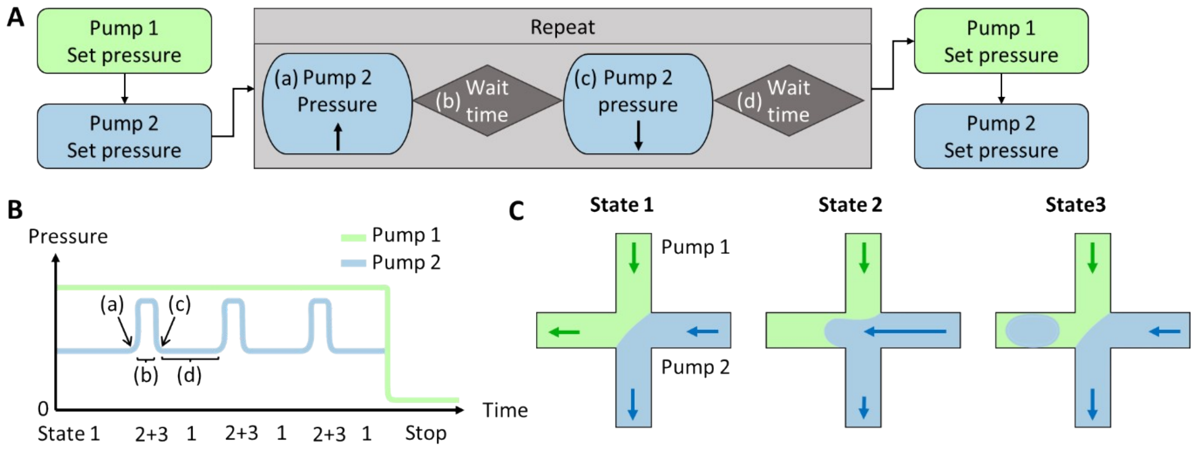

Figure S 2: The pressure program closely mirrors that of Figure 2. Initially, state 1 is set, followed by the configuration of pressure pulsing. During this process, the pressure of pump 2 (discontinuous phase) is raised (a). Subsequently, after a defined waiting period (b), the pressure of pump 2 is returned to its initial value (c). After a waiting period (d), multiple cycles can be executed to generate additional droplets, as seen in states 2 and 3. Finally, the pump pressures can be adjusted differently, such as setting them to ambient pressure to cease droplet generation.

Table S 1: This protocol is specifically designed for the yeast-catalysed reaction presented in Figure 5. An ambient pressure of 0 mbar is described by the relative pressure. Please refer to Figure 2 or S2 for the corresponding labels (a) to (d). The values of (a) to (d) are repeated five times.

	Set pressure	a) Pressure decrease	b) Waiting time	c) Pressure increase	d) Waiting time	Set pressure
Pump 1	320 mbar	80 mbar	150 ms	80 mbar	2 s	0 mbar
Pump 2	300 mbar					0 mbar
Pump 3	300 mbar					0 mbar

Droplet volume calculation

The droplets in PDMS channels were calculated using the approximate solutions (equation 1) of Musterd et al.[1] The PDMS channels are rectangular, and water does not wet the channel wall. A channel height (H) and width (W) of 100 µm was assumed. Droplet length (L) was determined using ImageJ (Fiji), and a reference slide was used for calibration.

$$V_{Droplet\ in\ PDMS} = \left[HW - (4-\pi)\left(\frac{2}{H}+\frac{2}{W}\right)^{-2}\right]\left(L-\frac{W}{3}\right) \quad (1)$$

The droplet in Figure S 3 is enclosed within a capillary with a 360 µm outer diameter (OD) and 100 µm inner diameter (ID). Consequently, it was approximated as a cylindrical base body with a 100 µm diameter (d=2r) corresponding to the ID. The height (h) was determined by measuring the microscopic image using ImageJ (Fiji), following calibration with a reference slide. For the rounded portions of the droplet, a spheroid was employed. One of the diameters was assumed to be 100 µm, while the second diameter (d=c_1 c_2) was calculated by subtracting the cylinder height (h) from the total droplet length. This relationship is expressed by Equation 2:

$$V_{Droplet\ in\ Capillary} = W_{Cylinder} + V_{Spheroid} = \pi r^2 h + 4/3\, \pi\, r \left(\frac{c_1\, c_2}{2}\right)^2 \quad (2)$$

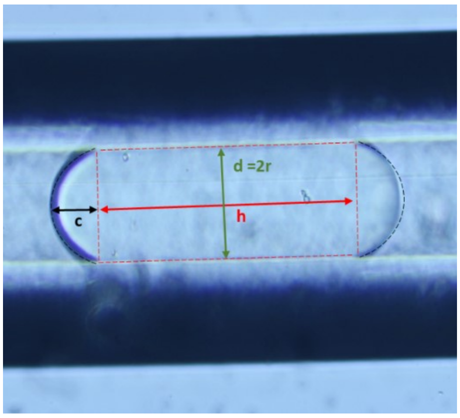

Figure S 3: Microscopic image of a droplet with the parameters necessary for calculating the droplet volume as per Equation 1.

Droplet on demand parameter screening

The parameters of hydrodynamic gating in PDMS chips were investigated. Droplet size and whether droplets were formed with each pulse were considered. The smallest droplets of 0.31 ± 0.02 nL were obtained at 450 mbar oil pressure, with several droplets per pulse. At 300 mbar the droplet size was 1.68 ± 0.07 nL with only 1 droplet per pulse (19/20 droplets per pulse). The optimum values for this configuration are approximately 300 mbar for the pressure of the discont. phase (FC-40) and cont. phase (water), a pressure pulse intensity of 8 mbar and a waiting time of 200-800 ms.

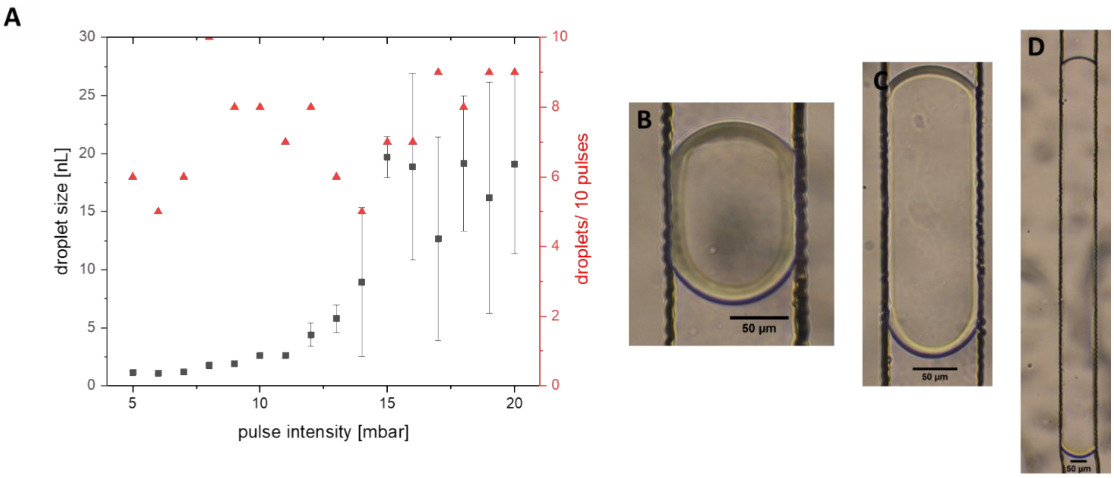

Figure S 4: Parameter screening of PDMS chips for pulse intensity. A standard deviation of less than 0.25 nL has been achieved up to 12 mbar. The smaller the pulse intensity, the smaller the droplet volume. The pulse was performed 20 times, and the droplets / 20 pulses describe whether droplets were produced with each pulse. The target of 1 droplet per pulse was only achieved at 8 mbar. One droplet each at pulse intensity C: 5 mbar, D: 11 mbar and E: 17 mbar were attached as microscopic images with a scale bar of 50 µm.

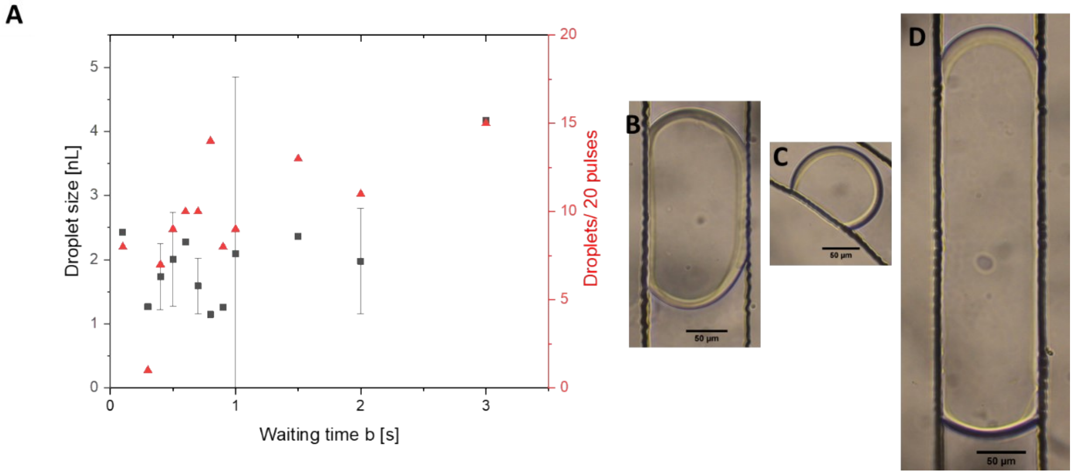

Figure S 5: Parameter screening of PDMS chips for waiting time b. The pressures of water and FC-40 were 300 mbar and the pressure was increased by 5 mbar during the pulse. The pulse was performed 20 times and the droplets / 20 pulses describe whether droplets were formed at each pulse. One droplet each at waiting time b C: 0.2 s, D: 0.8 s and E: 3 s were attached as microscopic images with a scale bar of 50 µm.

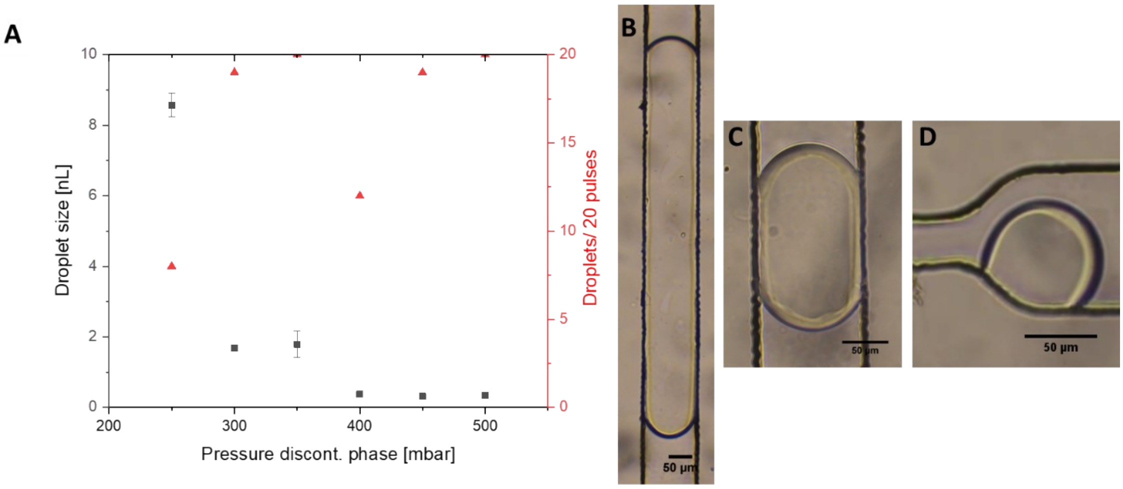

Figure S 6: Parameter screening of PDMS chips for oil pressure. The aqueous phase was adjusted so that a stable interphase was present. The aqueous phase was increased by 8 mbar and the waiting time b was 500 ms. The pulse was performed 20 times and the droplets / 20 pulses describe whether droplets were formed at each pulse. One droplet each at pressure C: 200 mbar, D: 350 mbar and E: 500 mbar were attached as microscopic images with a scale bar of 50 µm.

The glass chip setup was also tested. No significant differences in droplet size were found for the waiting time b and the pressure. If the waiting times were too short, fewer droplets were generated as pulses. Due to the greater back pressure generated in the capillaries, no differences in droplet size were found, even at higher pressures. At low pressures of 200 mbar only large plugs could be produced. The pulse intensity needed to be higher for droplet generation in glass chips. In addition, a comparison was conducted between the two glass chips depicted in Figure S 8. No significant differences in the average droplet size were observed. However, higher standard deviation values were recorded for chip 1. The microscopic images in Figure S 8 illustrates the droplet generation process, wherein the adaptation is considerably faster with chip 2. Additionally, chip 1 exhibited elevated pressure fluctuations. The difference in the waste capillary was not identified as a critical factor following the completion of several chip tests. Instead, the potential challenges may arise from the gluing of the capillaries, which can be addressed by utilising an alternative adhesive.

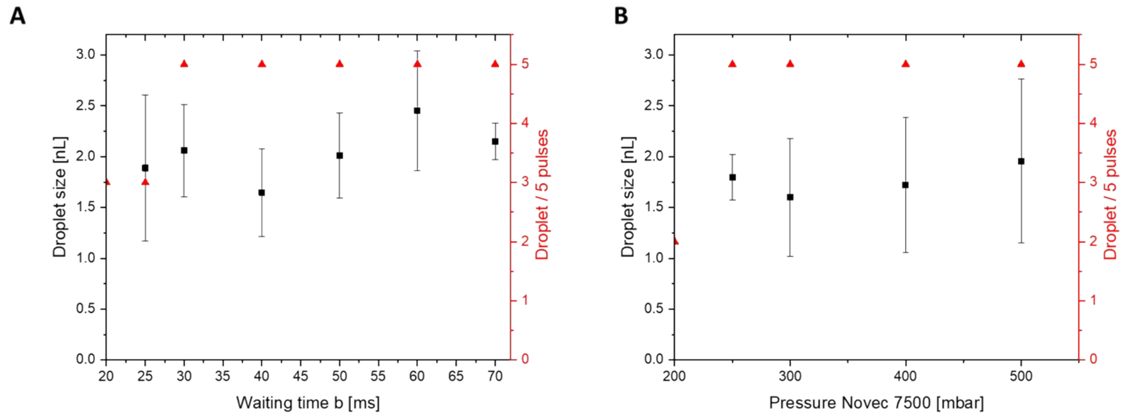

Figure S 7: Parameter screening of glass chips with A: waiting time b and B: pressure of Novec 7500. A: The pressure of the Novec 7500 was 500 mbar, the pressure of the water was 525/523 mbar and the pulse intensity was 50 mbar. B: The pulse intensity was 45 mbar and the waiting time b was 30 ms. The pulses were performed 5 times and no two droplets were detected per pulse. The new OxyGEN software from Fluigent was used for these experiments.

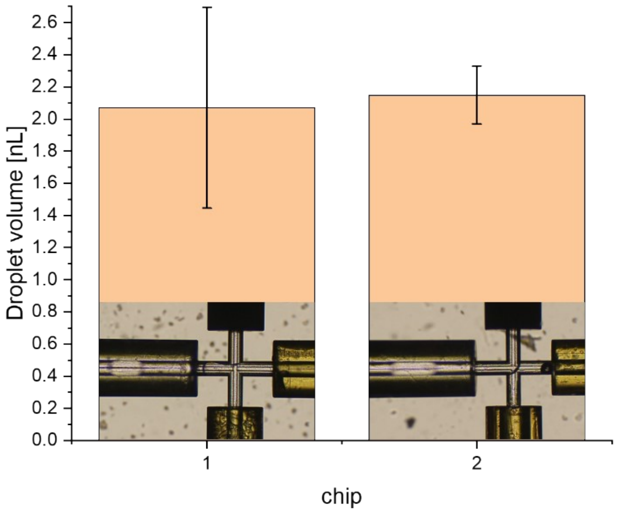

Figure S 8: A direct comparison of the average droplet volume of two glass chips, differing in the ID of the waste capillary: 100 µm for chip 1 and 200 µm for chip 2. Both chips were analysed on the same day with the following parameters: pressure oil 500 mbar; pressure water for chip 1: 500 mbar and for chip 2: 520 mbar; waiting time b 70 ms and pulse intensity 50 mbar. The new OxyGEN software from Fluigent was used for these experiments.

Setup for the generation of droplets

Figure S 9 depicts the setup for generating droplets with DoD using glass chips. In contrast, the setup for PDMS chips deviates, as it incorporates TFE capillaries with an outer diameter (OD) of 1/16" and an inner diameter (ID) of 300 µm. Figure S 10 illustrates the setup involving an upstream PEEK T-cross (VICI product no. C360QTPK4) for the cell-catalysed reaction. In this arrangement, two aqueous phases, comprising yeast cells and reactants, are thoroughly mixed and subsequently directed into the glass chip, where they encounter the continuous phase.

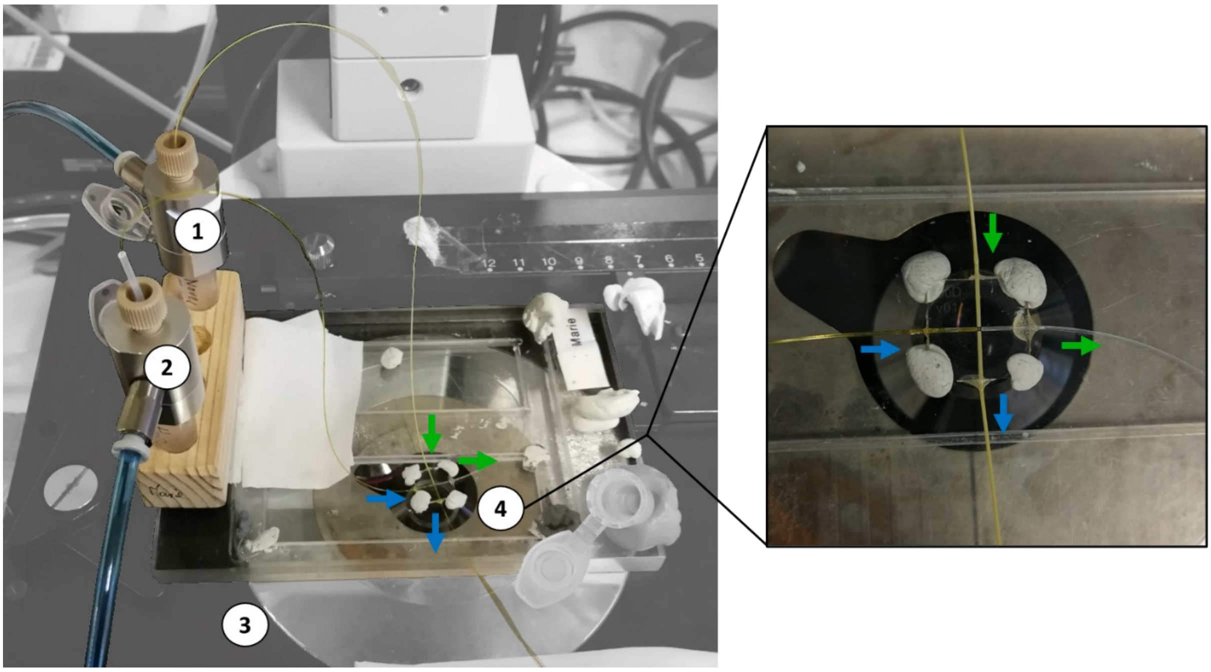

Figure S 9: Configuration for the Droplet-on-Demand (DoD) measurement setup using a glass chip (4). Fluigent pumps are connected to the reservoirs for the continuous phase (1, indicated by green arrows) and the discontinuous phase (2, indicated by blue arrows). This setup is positioned on a microscope (3) and can be adjusted in position using the x-y stage. The magnified view on the right displays the chip positioned above a 10× objective of the microscope.

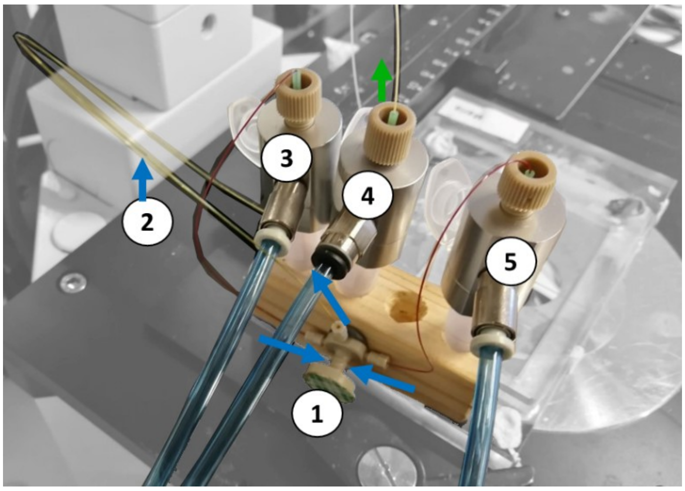

Figure S 10: Setup for the mixing the discont. phase (indicated by blue arrows) of yeast (3) and the reactant (5) using a PEEK T-cross (1). The resulting mixture is then introduced into the chip via the capillary (2). The continuous phase (4, indicated by a green arrow) is supplied directly to the chip, as demonstrated in Figure S 2.

Setup of a glass chip or HPFA capillary for MS measurements

For the MS measurements, the setup illustrated in Figure S 11 was employed. As depicted in Figure S 11B, the fused-silica glass chip, or an HPFA capillary featuring droplets positioned between the emitter and a capillary connected by the Nemesys syringe pump, was utilised. The generation or transition of droplets was closely monitored using a portable microscope. Variations in the ESI spray were monitored through a digital microscope (Andonstar; AD 407). If necessary, the parameters described in Table S2 can be adjusted. Furthermore, the height of the emitter capillary could be fine-tuned to align with the triple tube sprayer. Ideally, the HPFA emitter capillary should slightly protrude from the metal capillary, with the sheath liquid enveloping the capillary.

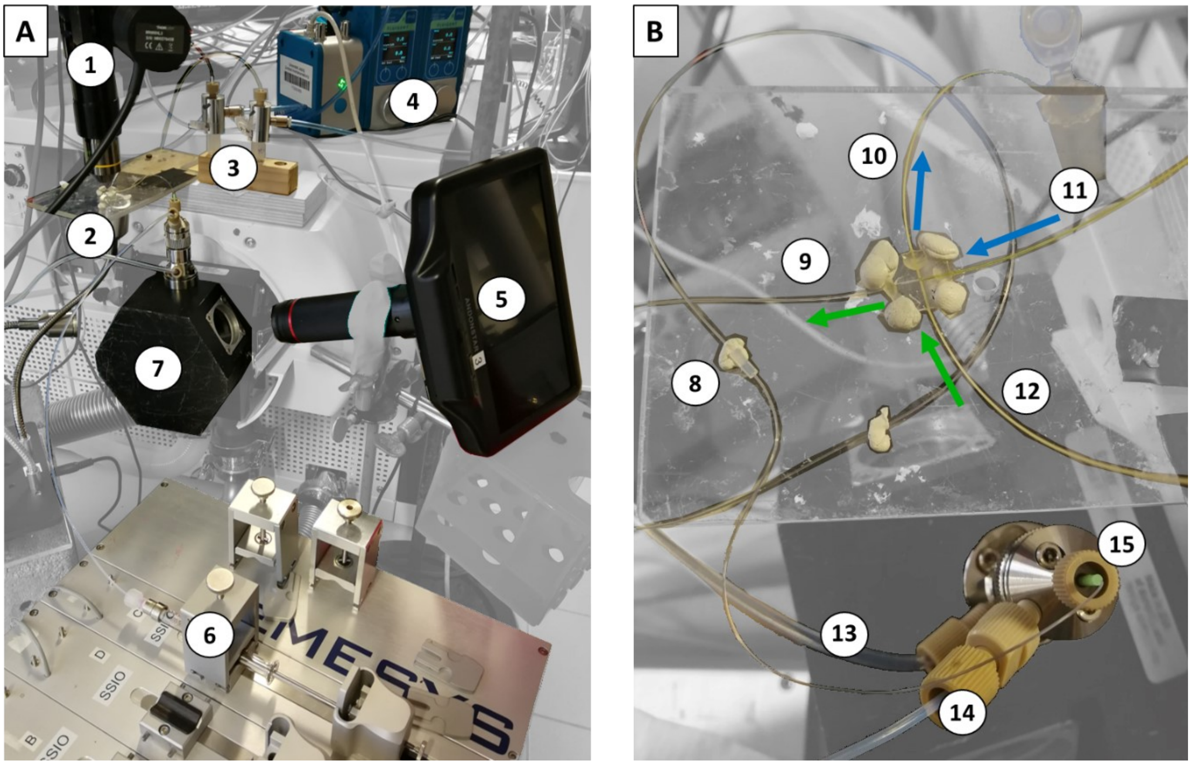

Figure S 11: A: Setup for droplet detection in ESI-MS (Agilent TQ; 6495 LC/TQ) using Agilent's triple tube sprayer. (1) Portable microscope; (2) glass chip; (3) continuous and discontinuous phases in their reservoir; (4) Fluigent pump; (5) digital microscope; (6) Nemesys syringe pump and (7) ESI chamber. B: Presents an enlarged view of the chip assembly and the triple tube sprayer. (8) TFE connector; (9) outlet to the droplet capillary; (10) outlet to the waste capillary; (11) inlet of the discontinuous phase; (12) Inlet of the continuous phase; (13) nebuliser gas; (14) sheath liquid and (15) triple tube sprayer.

Table S 2: MS parameters utilised for the measurement of the yeast-catalysed reaction, configured explicitly on the Agilent TQ MS.

**MS-Parameter**	**Value**
Gas temperature	100 °C
Gas flow	11 L/min
Nebuliser gas	5 psi
Voltage	- 4000 V
Collision energy	7 mV
Dwell time	2 ms

Storage capillary

Short HPFA capillary segments were employed to accommodate multiple droplets positioned side by side. These capillaries can remain unsealed for 1-2 hours, as depicted in Figure S 12. If extended storage was necessary, the capillaries were sealed with plugs at both ends.

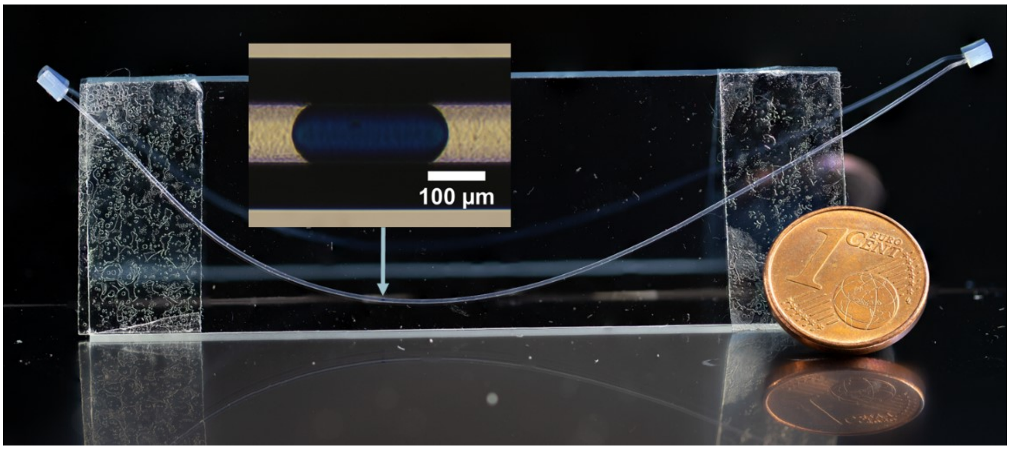

Figure S 12: An HPFA capillary (360 µm OD; 100 µm ID) containing a tiny blue droplet indicated by the blue arrow and a microscopic image of the droplet. TFE connecting capillaries (1/16" OD; 300 µm ID) are affixed to both capillary ends. The image was generated using focus stacking (Adobe Photoshop).

Mass trace evaluation

The mass trace of Figure 3 has been compared in Figure S 13 using the droplet volume of the signal width and the signal height. Normalisation to the highest value is used to improve comparability. The width of the MS signal corresponds better to the droplet volume than the height because of the time the droplet was sprayed. Therefore, there is a correlation between the width of the signal, the length of the droplet, and the flow.

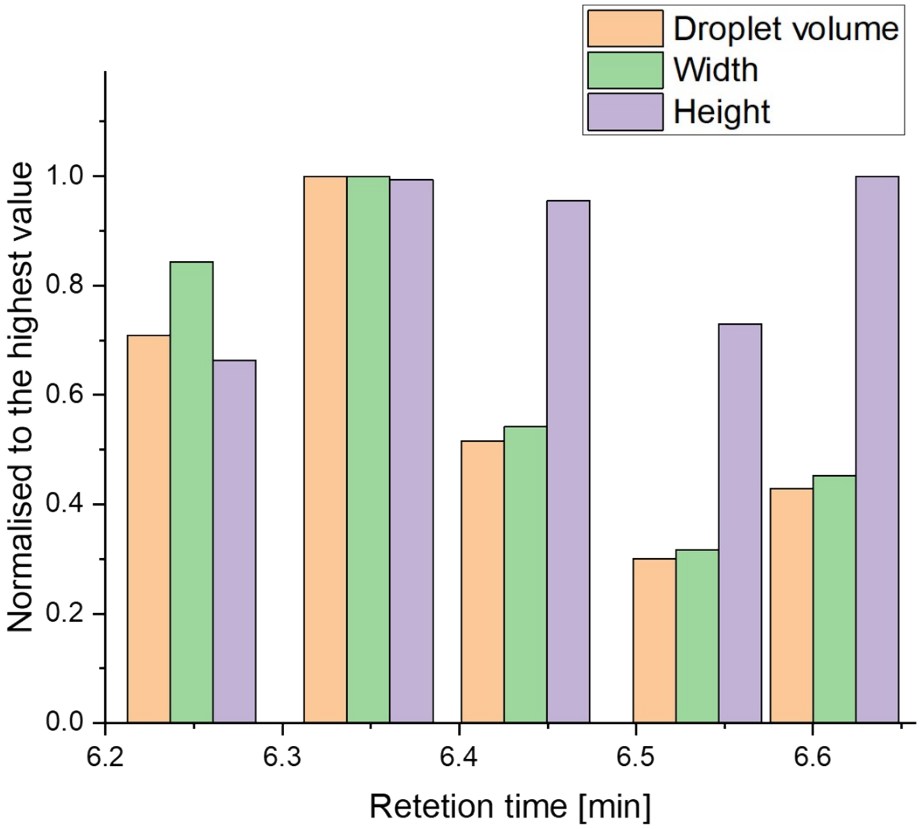

Figure S 13: Histogram comparison of the droplet volume (yellow) and the MS signal by width (green) and height (purple). For a better overview, the retention times of the 5 MS signals of the 5 droplets were plotted on the X-axis. The different values of the y-axis have been normalised by the highest value, so that the highest value is 1.

Figure S 14 illustrates a mass trace of a droplet surrounded by the continuous phase. The signal profile does not conform to a Gaussian distribution, as typically observed in chromatograms, due to the absence of diffusion into the

continuous phase. Consequently, the area under the droplet signal depends on the droplet's size. Conversely, the signal's peak intensity is notably influenced by the ESI spray and, thus, exhibits significant variability. Nonetheless, signal height is a more reliable metric for quantification and has been utilised in previous publications.[2,3]
In the context of droplets, which essentially serve as a direct injection of a reaction mixture, this publication determined the mean value of the signal using a custom MATLAB program. The calibration line's signal limits were defined by identifying the inflexion point of the internal standard (IS), a process that was further verified manually. The mean and standard deviation of the IS, analyte, and reactant signals were computed within the range delineated by the IS's inflexion points. The Grubbs test was then used to check for outliers.[4] For the yeast-catalysed reaction, the signal start and end values were manually ascertained using Agilent evaluation software (Qualitative Analysis 10.0). These signal boundaries are shaded in grey in Figure S 14. Subsequently, these start and end values were incorporated into the MATLAB script in place of the inflexion points, and the mean signal value was calculated as previously described.

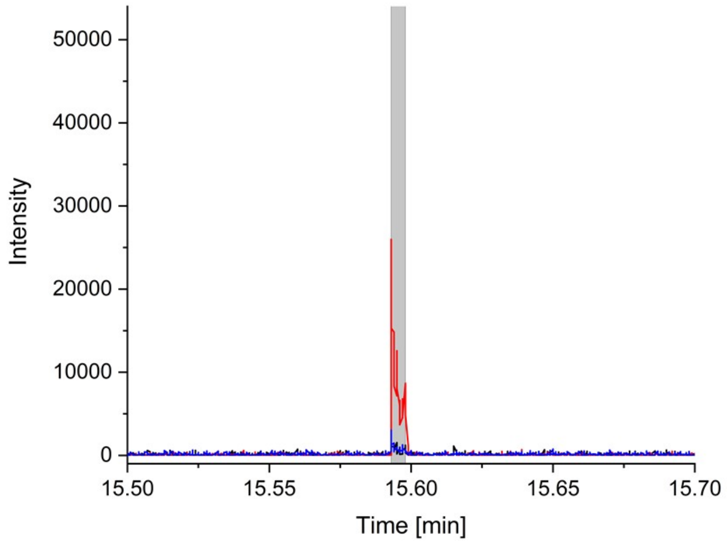

Figure S 14: Mass trace of a droplet derived from the data presented in Figure 5. The shaded grey region represents the analysis window used for computing the mean signal value.

Calculation of the LOD and LOQ

The following equations 3 and 4 were used to calculate the limit of detection (LOD) and limit of quantitation (LOQ) as recommended by the International Committee on Harmonization (ICH),[5] where SD is the standard deviation at x_0 and m is the slope of the calibration curve in Figure 5B.

$$LOD = 3.3\ SD/m \quad (3)$$
$$LOQ = 10\ SD/m \quad (4)$$

Mass traces of additional capillaries with droplets

In the case of the yeast-catalysed reaction depicted in Scheme 1, several additional capillaries were filled with a small number of droplets using the Droplet-on-Demand (DoD) technique. These capillaries were subsequently subjected to analysis via ESI/MS, enabling the identification and quantification of multiple yeast cells, as illustrated in Figure 5. The corresponding mass traces for these yeast cell-containing droplets are presented in Figure S 15 and S14. The data evaluation followed the procedures detailed in the 'Mass Trace Evaluation' section, and the resulting values are consolidated in Table S3. The data were used to plot droplet volume against EHB concentration, as shown in Figure S 17. However, no correlation was found.

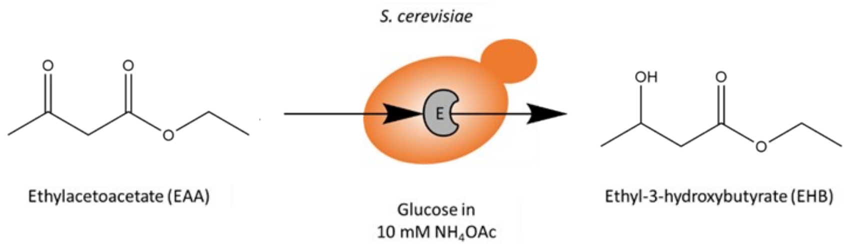

Scheme 1: Yeast-catalysed reaction from a ketoester (EAA) to ethyl-3-hydroxybutyrate (EHB).

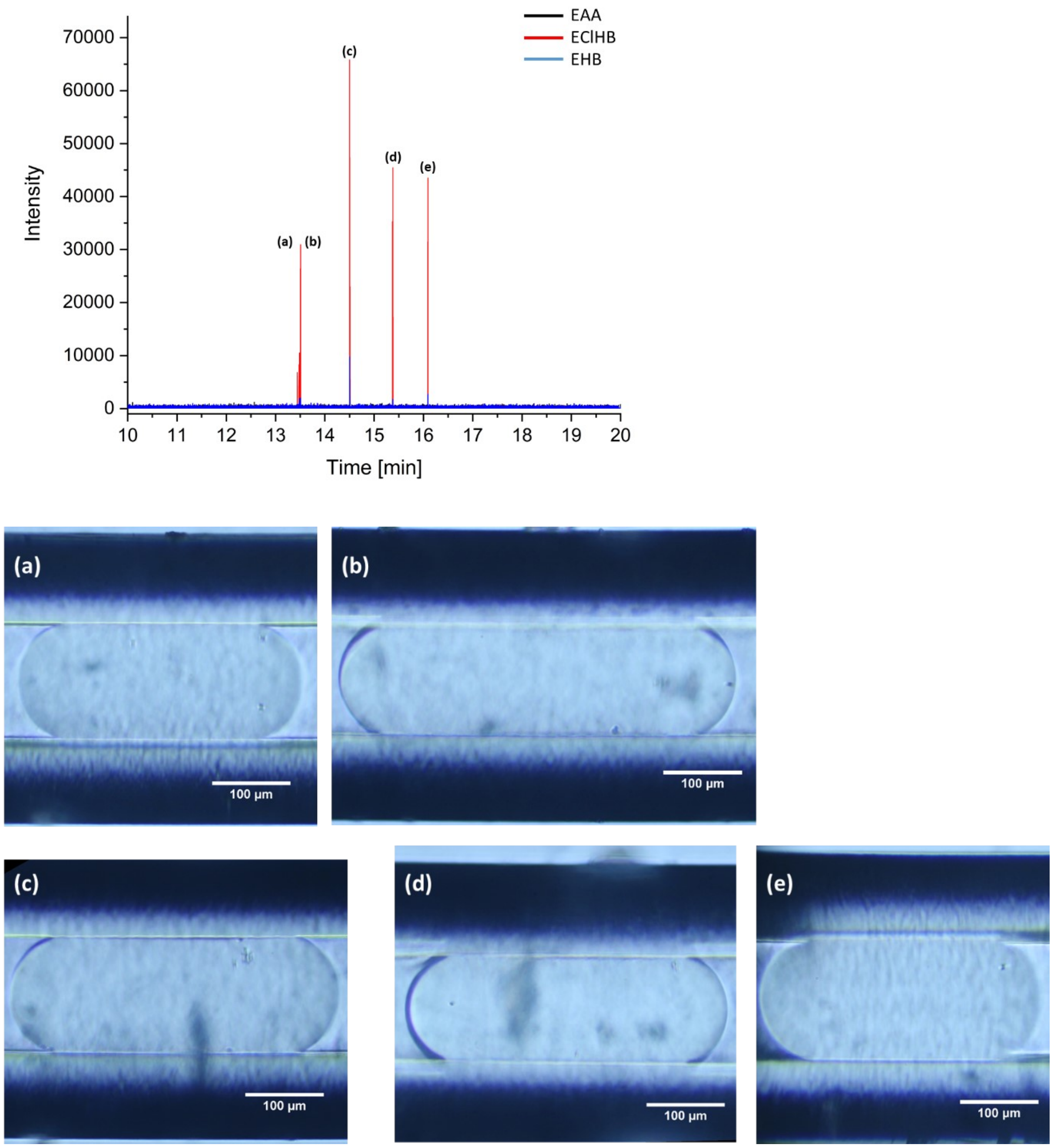

Figure S 15: Mass trace of five droplets generated using the Droplet-on-Demand (DoD) technique. In the mass trace, the initial signal comprises two closely spaced droplets. The microscopic images of the droplets are labelled (a) to (e) and correspond to the respective signals. Table S3 provides information on the associated cell number, droplet volume, and production formation rate.

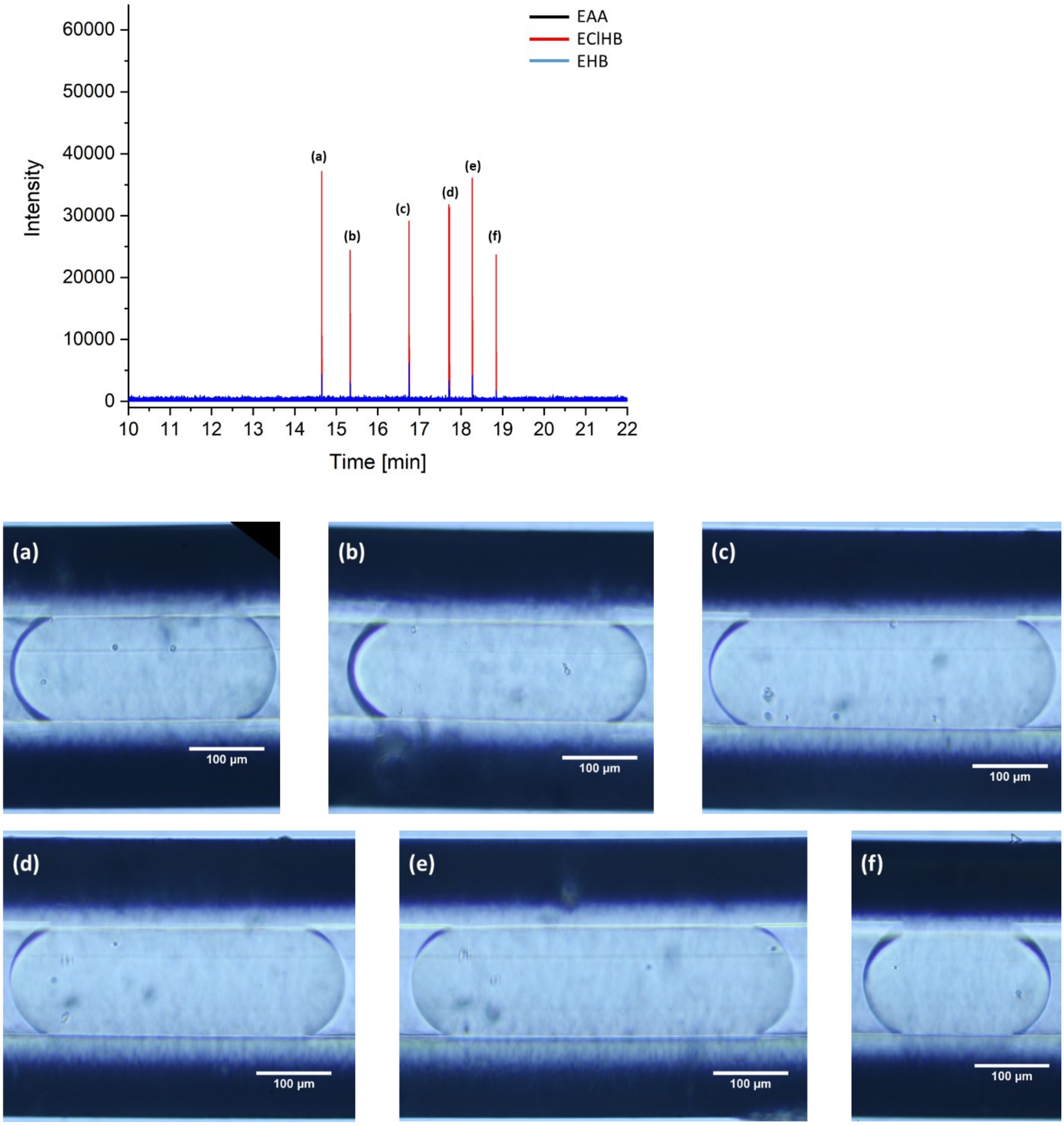

Figure S 16: Mass trace of six droplets generated by DoD. A second droplet was created during a pulse. The microscopic images of the droplets are marked as (a) to (f) and can be correlated with the corresponding signals. Table S3 presents data on the cell number, droplet volume, and production formation rate associated with these droplets.

Table S 3: For the yeast-catalysed reaction, the concentration of the product EHB was determined via calibration. The images from Figures 5, S15 and S16 and a procedure similar to that in Video 2 were used to determine the number of cells in the droplets and the corresponding droplet volumes. This information enabled the calculation of the product formation rate, given that the yeast-catalysed reaction had a duration of approximately 20 hours for each droplet.

Figure		Product EHB conc. [µM]	Cell Number	Droplet volume [nL]	Product formation rate [fmol/cell/h]
5	(a)	0.319	1	1.6	0.026
	(b)	0.769	1	2.0	0.077
	(c)	0.586	2	2.0	0.029
	(d)	0.779	9	4.6	0.020
	(e)	0.652	5	4.7	0.031

S15	(a)	1.175	3	2.6	0.051
	(b)	0.341	3	3.7	0.021
	(c)	0.963	6	3.0	0.024
	(d)	0.242	3	3.0	0.012
	(e)	0.536	1	2.5	0.067
S16	(a)	0.875	4	2.6	0.028
	(b)	0.921	4	2.9	0.033
	(c)	1.365	7	3.3	0.032
	(d)	0.867	5	3.2	0.028
	(e)	1.164	7	3.7	0.031
	(f)	0.607	3	1.7	0.052

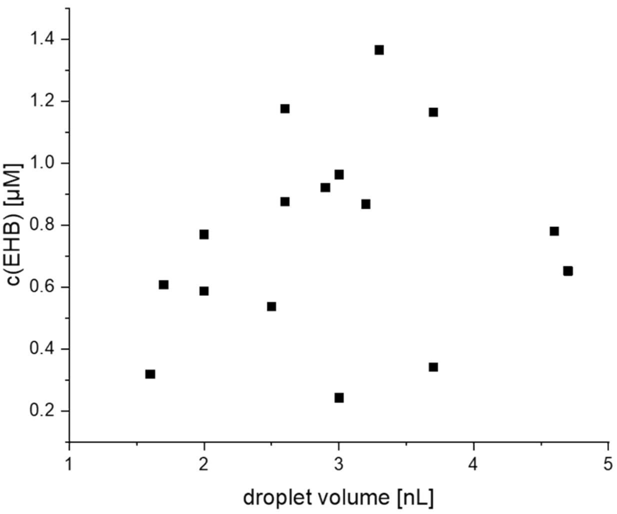

Figure S 17: Plot of droplet volume versus concentration of product EHB from the droplets of the MS signals in Figures 5, S15 and S16.

Droplet Generation Videos

Video 1: In this video, the formation of the droplets is visualised as shown in the figure 5.

Droplet Examination Video

Video 2: In this video, droplets containing yeast cells have been examined. Droplet 1 contains three cells in the first focal plane and four cells in the second. Droplet 2 contains three cells in the first focal plane and six cells in the second focal plane. Droplet 3 contains only two cells.

Capillary Connection Video

Video 3: This video captures the flow of droplets passing through the HPFA capillary connector.

References

1 M. Musterd, V. van Steijn, C. R. Kleijn and M. T. Kreutzer, Calculating the volume of elongated bubbles and droplets in microchannels from a top view image, *RSC Adv.*, 2015, **5**, 16042–16049.

2 S. Sun, T. R. Slaney and R. T. Kennedy, Label free screening of enzyme inhibitors at femtomole scale using segmented flow electrospray ionization mass spectrometry, *Analytical chemistry*, 2012, **84**, 5794–5800.

3 X. W. Diefenbach, I. Farasat, E. D. Guetschow, C. J. Welch, R. T. Kennedy, S. Sun and J. C. Moore, Enabling Biocatalysis by High-Throughput Protein Engineering Using Droplet Microfluidics Coupled to Mass Spectrometry, *ACS omega*, 2018, **3**, 1498–1508.

4 F. W. Küster and A. Thiel, *Rechentafeln für die chemische Analytik*, de Gruyter, Berlin, 106th edn., 2008.

5 International Council for Harmonisation (ICH), *ICH guideline Q2(R2) on validation of analytical procedures*, European Medicines Agency, 2022.
